# Supplementary material for: Heat adaptation of phage T7 under an extended genetic code
Source: Virus Evol. 2021 Dec 1;7(2):veab100. doi: 10.1093/ve/veab100 (PMC8923235; doi:10.1093/ve/veab100)
Supplement: veab100_Supp [file veab100_supp.zip › Sequencing_Analysis.zip!/Sequencing_Analysis/S1/evidence/INS_8.html]

BRESEQ :: Evidence


| Predicted mutation | | | | | | |
| --- | --- | --- | --- | --- | --- | --- |
| evidence | seq id | position | mutation | annotation | gene | description |
| JC | NC\_001604.1 | 27,769 | (ATATCTGGCGCT)_1→2_ | intergenic (–/–) | – / – | –/– |

| New junction evidence | | | | | | | | | | | |
| --- | --- | --- | --- | --- | --- | --- | --- | --- | --- | --- | --- |
|  | | seq id | position | reads (cov) | reads (cov) | score | skew | freq | annotation | gene | product |
| \* | ? | NC\_001604.1 | = 27753 | 15 (0.000) | 3703 (0.920) +CGCTATATCTGG | 393/544 | 0.0 | 99.6% | intergenic (–/–) | *–/–* | –/– |
|  | ? | NC\_001604.1 | 27754 = | 18 (0.000) |  |  |  |  | intergenic (–/–) | *–/–* | –/– |

Only 100 of 4091 total aligned reads displayed.  

`TCTAGCTATCGGTGGTAACTGCGGGGACCAGTGCTGGTTCGTTACGAGCGACCAAGTGTGGCGACTTAGTGGAAAGGCTAAGCGAAAGTTCCGTAAGTTAATCATGGAGTATCGCGATAAGATGCTTGAGAAGTATGATACTCTTTGGAATTACGTATGGGTAGGCAATACGTCCCACATTCGTTTCCTCAAGACTATCGGTGCGGTATTCCATGAAGAGTACACACGAGATGGTCAATTTCAGTTATTTACAATCACGAAAGGAGGATAACCATA‑‑TGTGTTGGGCAGCCGCAATACCTATCGCTATATCTGGCGCTCAGGCTATCAGTGGTCAGAACGCTCAGGCCAAAATGATTGCCGCTCAGACCGCTGCTGGTCGTCGTCAAGCTATGGAAATCATGAGGCAGACGAACATCCAGAATGCTGACCTATCGTTGCAAGCTCGAAGTAAACTTGAGGAAGCGTCCGCCGAGTTGACCTCACAGAACATGCAGAAGGTCCAAGCTATTGGGTCTATCCGAGCGGCTATCGGAGAGAGTATGCTTGAAGGTTCCTCAATGGACCGCATTAAGCGAGTCACAGAAGGACAGTTCATTCGGGA  >  NC_001604.1/27453‑28053`  

                                                                                                                                                                                                                                                                                                                                                                                                                                                                                                                                                                                                                             
     `cTATCGGTGGTAACTGCGGGGACCAGTGCTGGTTCGTTACGAGCGACCAAGTGTGGCGACTTAGTGGAAAGGCTAAGCGAAAGTTCCGTAAGTTAATCATGGAGTATCGCGATAAGATGCTTGAGAAGTATGATACTCTTTGGAATTACGTATGGGTAGGCAATACGTCCCACATTCGTTTCCTCAAGACTATCGGTGCGGTATTCCATGAAGAGTACACACGAGATGGTCAATTTCAGTTATTTACAATCACGAAAGGAGGATAACCATA‑‑TGTGGTGGGCAGCCGCAATACCTATcgctt                                                                                                                                                                                                                                                                                                         >  1:668746‑M1/1‑296 (MQ=255)`  
            `tggtAATTGCGGGGACCAGTGATGGTTGGTTACGAGCGACCAAGTGTGGCGACTTAGTGGAAAGGCTAAGCGAAAGTTGCGTAAGTTAATCATGGAGTATCGCGATAAGATGCTTGAGAAGTATGATACTCTTTGGAATTACGTATGGGTAGGCAATACGTCCCACATTCGTTTCCTCAAGACTATCGGTGCGGTATTCCATGAAGAGTACACACGAGATGGTCAATGTCAGTTATTTACAATCACGAAAGGAGGATAACCATA‑‑TGTGTTGGGCAGCCGCAATACCTATcgctatatctgg                                                                                                                                                                                                                                                                                                  <  1:471072‑M1/301‑13 (MQ=255)`  
            `tggtAACTGCGGGGACCAGTGCTGGTTCGTTACGAGCGACCAAGTGTGGCGACTTAGTGGAAAGGCTAAGCGAAAGTTCCGTAAGTTAATCATGGAGTATCGCGATAAGATGCTTGAGAAGTATGATACTCTTTGGAATTACGTATGGGTAGGCAATACGTCCCACATTCGTTTCCTCAAGACTATCGGTGCGGTATTCCATGAAGAGTACACACGAGATGGTCAATTTCAGTTATTTACAATCACGACAGGAGGATAACCATA‑‑TGTGTTGGGCATCCGCAATACCTATctatatatcctg                                                                                                                                                                                                                                                                                                  >  1:104790‑M1/1‑289 (MQ=255)`  
             `ggtAACTGCGGGGACCAGTGCTGGTTCGTTACGAGCGACCAAGTGTGGCGACTTAGTTGAAAGGCTAAGCGAAAGTTCCGTAAGTTAATCATGGAGTATCGCGATAAGATGCTTGAGAAGTATGATACTCTTTGGAATTACGTATGGGTAGGCAATACGTCCCACATTCGTTTCCTCAAGACTATCGGTGCGGTATTCCATGAAGAGTACACACGAGATGGTCAATTTCAGTTATTTACAATCACGAAAGGAGGATAACCATA‑‑TGTGTTGGGCAGCCGCAATACCTATcgctatatctctt                                                                                                                                                                                                                                                                                                 >  1:128247‑M1/1‑288 (MQ=255)`  
               `gaacTGCGGGGACCAGGGCTGGTTCGTTACGAGCGACCAAGTGTGGCGACTTAGTGGAAAGGCTAAGCGAAAGTTCCGTAAGTTAATCATGGAGTATCGCGATAAGATGCTTGAGAAGTATGATACTCTTTGGAATTACGTATGGGTAGGCAATACGTCCCACATTCGTTTCCTCAAGACTATCGGTGCGGTATTCCATGAAGAGTACACACGAGATGGTCAATTTCAGTTATTTACAATCACGAAAGGAGGATAATCATA‑‑TGTGTTGGGCAGCCGCAATACCTATcgctatatctggcgc                                                                                                                                                                                                                                                                                               <  1:611325‑M1/300‑16 (MQ=255)`  
                  `cTGCGGGGACCAGTGCT‑GTTCGTTACGAGCGACCAAGTGTGGCGACTTAGTGGAAAGGCTAAGCGAAAGTTCCGTAAGTTAATCATGGAGTATCGCGATAAGATGCTTGAGAAGTATGATACTCTTTGGAATTACGTATGGGTAGGCAATACGTCCCACATTCGTTTCCTCAAGACTATCGGTGCGGTATTCCATGAAGAGTACACACGAGATGGTCAATTTCAGTTATTTACAATCACGAAAGGAGGATAACCATA‑‑TGTGTTTGGCAGCCGCAATACCTATcgctatatctggcgctata                                                                                                                                                                                                                                                                                           >  1:185388‑M1/1‑282 (MQ=255)`  
                     `aagagacaggtgctggttcGTTACGAGCGACCAAGTGTGGCGACTTAGTGGAAAGGCTAAGCGAAAGTTCCGTAAGTTAATCATGGAGTATCGCGATAAGATGCTTGAGAAGTATGATACTCTTTGGAATTACGTATGGGTAGGCAATACGTCCCACATTCGTTTCCTCAAGACTATCGGTGCGGTATTCCATGAAGAGTACACACGAGATGGTCAATTTCAGTTATTTACAATCACGAAAGGAGGATAACCATA‑‑TGTGTTGGGCAGCCGCAATACCTATcgctatatctggcgctatatc                                                                                                                                                                                                                                                                                         <  1:416999‑M1/292‑22 (MQ=255)`  
                          `aCCAGTGCTGGTTCGTTACGAGCGACCAAGTGTGGCGACTTAGTGGAAAGGCTAAGCGAAAGTTCCGTAAGTTAATAATGGAGTATCGCGATAAGATGCTTGAGAAGTATGATACTCTTTGGAATTACGTATGGGTAGGCAATACGTCCCACATTCGTTTCCTCAAGGCTATCGGTGCGGTATTCCATGAAGAGTACACACGAGATGGTCAATTTCAGTTATTTACAATCACGAAAGGAGGATAACCATA‑‑TGTGTTGGGCAGCCGCAATACCTATcgctatatctggcgctatatctggcg                                                                                                                                                                                                                                                                                    <  1:382985‑M1/301‑27 (MQ=255)`  
                           `ccAGTGCTGGTTCGTTACGAGCGACCAAGTGTGGCGACTTAGTGGAAAGGCTAAGCGAAAGTTCCGTAAGTTAATCATGGAGTATCGCGATAAGATGCTTGAGAAGTATGATACTCTTTGGAATTACGTATGGGTAGGCAATACGTCCCACATTCGTTTCCTCAAGACTATCGGTGCGGTATTCCATGAAGAGTACACACGAGATGGTCAATTTCAGTTATTTACAATCACGAAAGGAGGATAACCATA‑‑TGTGTTGGGCAGCCGCAATACCTATcgctatatctggcgctatatctggcgc                                                                                                                                                                                                                                                                                   >  1:483412‑M1/1‑274 (MQ=255)`  
                           `ccAGTGCGGGTTCGTTACGAGAGACCAAGTGTGGCGACTTAGTGGAAAGGCTAAGCGAAAGTTCCGTAAGTTAATCATGGAGTATCGCGATAAGATGCTTGAGAAGTATGATACTCTTTGGAATTACGTATGGGTAGGCAATACGTCCCACATTCGTTTCCTCAAGACTATCGGTGCGGTATTCCATGAAGAGTACACACGAGATGGTCAATTTCAGTTATTTACAATCACGAAAGGAGGATAACCATA‑‑TGTGTTGGGCAGCCGCAATACCTATcgctatatctggcgctatatctggcgc                                                                                                                                                                                                                                                                                   <  1:263158‑M1/301‑28 (MQ=255)`  
                           `aaggtgcTGGTTCGTTACGGGCGACCAAGTGTGGCGAATTAGTGGAAAGGCTAAGCGAAAGTTCCGTAAGTTAATCATGGAGTATCGCGATAAGATGCTTGAGAAGTATGAGACTCTTTGGAATTACGTATGGGTAGGCAATACGTCCCACATTCGTTTCCTCAAGACTATCGGTGCGGTATTCCATGAAGAGTACACACGAGATGGTCAATTTCAGTTATTTACAATCACGAAAGGAGGATAACCATA‑‑TGTGTTGGGCAGCCGCAATACCTATcgctatatctggcgctatatctggcgc                                                                                                                                                                                                                                                                                   <  1:647768‑M1/298‑28 (MQ=255)`  
                              `gTGCTGGTTCGTTACGAGCGACCAAGTGTGGCGACTTAGTGGAAAGGCTAAGAGAAAGTTCCGTAAGTTAATCATGGAGTATCGCGATAAGATGCTTGAGAAGTATGATACTCTTTGGAATTACGTATGGGTAGGCAATACGTCCCACATTCGTTTCCTCACGACTATCGGTGCGGTATTCGATGAAGAGTACACACGAGCTGTTCAATTTCAGCTATTTACAATCACGAAAGGAGGATAACCATA‑‑TTTGTTGGGCCGTCGCAATCCCTCTcgctatatctggcgatatttctggcgctca                                                                                                                                                                                                                                                                                >  1:459791‑M1/1‑271 (MQ=255)`  
                                       `cGTTACGAGCGACCAAGTGTGGCGACTTAGTGGAAAGGCTAAGCGAAAGTTCCGTAAGTTAATCATGGAGTATCGCGATAAGATGCTTGAGAAGTATGATACTCTTTGGAATTACGTATGGGTAGGCAATACGTCCCACATTCGTTTCCTCAAGACTATCGGTGCGGTATTCCATGAAGAGTACACACGAGATGGTCAATTTCAGTTATTTACAATCACGAAAGGAGGATAACCATA‑‑TGTGTTGGGCAGCCGCAATACCTATcgctatatctggcgctatatctggcgctcaggctatcag                                                                                                                                                                                                                                                                       >  1:529558‑M1/1‑262 (MQ=255)`  
                                               `gCGACCAAGTGTGGCGACTTAGTGGAAAGGCTAAGCGAAAGTTCCGTAAGTTAATCATGGAGTATCGCGATAAGATGCTTGAGAAGTATGATACTCTTTGGAATTACGTATGGGTAGGCAATACGTCCCACATTCGTTTCCTCAAGACTATCGGTGCGGTATTCCATGAAGAGTACACACGAGATGGTCAATTTCAGTTATTTACAATCACGAAAGGAGGATAACCATA‑‑TGTGTTGGGCAGCCGCAATACCTATcgctatatctggcgctatatctggcgctcaggctatcagtggtcaga                                                                                                                                                                                                                                                               <  1:421598‑M1/301‑48 (MQ=255)`  
                                                               `aCTTAGTGGAAAGGCTAAGCGAAAGTTCCGTAAGTTAATCATGGAGTATCGCGATAAGATGCTTGAGAAGTATGATACTCTTTGGAATTACGTATGGGTAGGCAATACGTCCCACATTCGTTTCCTCAAGACTATCGGTGCGGTATTCCATGAAGAGTACACACGAGATGGTTAATTTCAGTTATTTACAATCACGAAAGGAGGATAACCATA‑‑TGTGTTGGGCAGCCGCCATACCTATcgctatatctggcgctatatctggcgatcaggctatcagtggtcagaacgcctttctcttatt                                                                                                                                                                                                                                               >  1:476544‑M1/1‑238 (MQ=255)`  
                                                                              `tAAGCGAAAGTTCACTAAGTGAATCATGGAGTATAGCGATAAGATGCTTGAGAAGTATGATCCTCTTTGGAATTACGTATGGGTAGGCAATACGTCCCACATTCGTTTCCTCAAGACTATCGGTGCGGTATTCCATGAAGAGTACACACTAGATGGTCAATTTCAGTTATTTACAATCACGAAAGGAGTATAACCATA‑‑TGTGTTGGGCAGCCGCAATACCTATcgctatatctggcgctatatctggcgctcaggctatcagtggtcagaacgctcaggccaaaatgattgccgctcagac                                                                                                                                                                                                                                <  1:69951‑M1/301‑79 (MQ=255)`  
                                                                                         `gacagaagttaaTCATGGAGTATCGCGATAAGATGCTTGAGAAGTATGATACTCTTTGGAATTACGTATGGGTAGGCAATACGTCCCACATTCGTTTCCTCAAGACTATCGGTGCGGTATTCCATGAAGAGTACACACGAGATGGTCAATTTCAGTTATTTACAATCACGAAAGGAGGATAACCATA‑‑TGTGTTGGGCAGCAGCAATACCTATcgctatatctggcgctatatctggcgctcaggctatcagtggtcagaacgctcaggccaaaatgattgccgctcagaccgctgctggtc                                                                                                                                                                                                                     <  1:549303‑M1/296‑90 (MQ=255)`  
                                                                                                   `aaTCATGGAGTATCGCGATAAGATGCTTGAGAAGTATGATACTCTTTGGAATTACGTATGGGTAGGCAATACGTCCCACATTCGTTTCCTCAAGACTATCGGTGCGGTATTCCATGAAGAGTACACACGAGATGGTCAATTTCAGTTATTTACAATCACGAAAGGAGGATAACCATA‑‑TGTGTTGGGCAGCCGCAATACCTATcgctatatctggcgctatatctggcgctcaggctatcagtggtcagaacgctcaggacaaaatgattgccgctcagaccgctgctggtcgtcgtcaagc                                                                                                                                                                                                           <  1:466143‑M1/301‑100 (MQ=255)`  
                                                                                                   `aaTCATGGAGTATCGCGATAAGATGCTTGAGAAGTATGATACTCTTGGGAATTACGTATGGGTAGGCAATACGTCCCACATTCGTTTCCTCAAGACTATCGGTGCGGTATTCCATGAAGAGTACACACGAGATGGTCAATTTCAGTTATTTACAATCACGAAAGGAGGATAACCATA‑‑TGTGTTGGGCAGCCGCAATACCTATcgctatatctggcgctatatctggcgctcaggctatcagtggtcagaacgctcaggccaaaatgattgccgctcagaccgctgctggtcgtcgtcaagc                                                                                                                                                                                                           <  1:274923‑M1/301‑100 (MQ=255)`  
                                                                                                    `aTCATGGAGTATCGCGATAAGATGCTTGAGAAGTATGATACTCTTTGGAATTACGTATGGGTAGGCAATACGTCCCACATTCGTTTCCTCAAGACTATCGGTGCGGTATTCCATGAAGAGTACACACGAGATGGTCAATTTCAGTTATTTACAATCACGAAAGGAGGATAACCATA‑‑TGTGTTGGGCAGCCGCAATACCTATcgctatatctggcgctatatctgtcgctcaggctatcagtggtcagaacgctcaggccaatatgattgccgctcagaccgctgctggtcgtcggcaagct                                                                                                                                                                                                          >  1:599829‑M1/1‑201 (MQ=255)`  
                                                                                                    `aTCATGGAGTATCGCGATAAGATGCTTGAGAAGTATGATACTCTTTGGAATTACGTATGGGTAGGCAATACGTCCCACATTCGTTACCTCAAGACTATCGGTGCGGTATTCCATGAAGAGTACACACGAGATGGTCAATTTCAGTTATTTACAATCACGAAAGGAGGATAACCATA‑‑TGGTTTGGGCAGCCGCAATACCTACcgcaatatctggcgctatatctggcgcacggcctttaagtggtagaaaccccaaggccaaattgatggccgctcagcccgctcttggtctccgcaaacct                                                                                                                                                                                                          >  1:303365‑M1/1‑201 (MQ=255)`  
                                                                                                                            `cTTGAGAAGTATGATACTCTTTGGAATTACGTATGGGTAGGCAATACGTCCCACATTCGTTTCCTCAAGACTATCGGTGCGGTATTCCATGAAGAGTACACACGAGATGGTCAATTTCAGTTATTTACAATCACGAAAGGAGGATAACCATATGTGTGTTGGGCAGCCGCAATACCTATcgctatatctggcgctatatctggcgctcaggctatcagtggtcagaacgctcaggccaaaatgattgccgctcagaccgctgctggtcgtcgtcaagctatggaaatcatgcggcagacga                                                                                                                                                                                    >  1:147532‑M1/1‑179 (MQ=255)`  
                                                                                                                               `gagaAGTATGATACTCTTTGGAATTACGTATGGGTAGGCAATACGTCCCACATTCGTTTCCTCAAGACTATCGGTGCGGTATTCCATGAAGAGTACACACGAGATGGTCAATTTCAGTTATTTACAATCACGAAAGGAGGATAACCATA‑‑TGTGTTGGGCAGCCGCAATACCTATcgctatatctggcgctatatctggcgctcaggctatcagtggtcagaacgctcaggccaaaatgatggccgctcagaccgctgctggtcgtcgtcaagctatggaaatcatgaggcagacgaacatc                                                                                                                                                                               >  1:488908‑M1/1‑174 (MQ=255)`  
                                                                                                                                    `gTATGATACTCTTTGGAATTACGTATGGGTAGGCAATACGTCCCACATTCGTTTCCTCAAGACTATCGGTGCGGTATTCCATGAAGAGTACACACGAGATGGTCAATTTCAGTTATTTACAATCACGAAAGGAGGATAACCATA‑‑TGTGTTGGGCAGCCGCAATACCTATCGCTATATCTGGCGCTCAGGCTATCAGTGGTCAGAACGCTCAGGCCAACATGATTGCCGCTCAGACCGCTGCTGGTCGTCGTCAAGCTATGGAAATCATGAGGCAGACGAACATCCAGAATGCCGACCTATc                                                                                                                                                                          >  1:173744/1‑301 (MQ=255)`  
                                                                                                                                      `aTGATACTCTTTGGAATTACGTATGGGTAGGCAATACGTCCCACATTCGTTTCCTCAAGACTATCGTTGCGGTATTCCATGAAGAGTACACACGAGATGTTCAATTTCAGTTATTTACAATCACGAAAGGAGGATAACCCTA‑‑TGTGTTGGGCAGCCGCAATACCTATcgctatatctggcgctatatctggcgctcaggctatcagtggtcagaacgctcaggccaaaatgattgccgctcagaccgctgctggtcgtcgtcaagctatggaaaccatgaggcagacgaacacccagattt                                                                                                                                                                        >  1:81415‑M1/1‑167 (MQ=255)`  
                                                                                                                                            `ctctTTGGAATTACGTATGGGTAGGCAATACGTCCCACATTCGTTTCCTCAAGACTATCGGTGCGGTATTCCATGAAGAGTACACACGAGATGGTCAATTTCAGTTATTTACAATCACGAAAGGAGGATAACCATA‑‑TGTGTTGGGCAGCCGCAATACCTATcgctatatctggcgctatatctggcgctcaggctatcagttgtcagaacgctcaggccaaaatgattgccgctcagaccgctgctggtcgtcgtcaagctatggaaatcatgaggcagacgaacatccagaatgctgacc                                                                                                                                                                  >  1:518485‑M1/1‑161 (MQ=255)`  
                                                                                                                                             `tctTTGGAATTACGTATGGGTAGGCAATACGTCCCACATTCGTTTCCTCAAGACTATCGGTGCGGTATTGCATGAAGAGTACACACGAGATGGTCAATTTCAGTTATTTACAATCACGAAAGGAGGATAACCATA‑‑TGTGTTGGGCAGCCGCAATACCTATcgctatatctggcgatatatctggcgctcatgctatcagtggtcagaacgctcaggccaaaatgattgccgttcagaccgctgctggtcgtcgtcaagctatggaaatcatgaggatgacgaacatccagaatgctgacct                                                                                                                                                                 >  1:617425‑M1/1‑160 (MQ=255)`  
                                                                                                                                              `ctTTGGAATTACGTATGGGTAGGCAATACGTCCCACATTCGTTTCCTCAAGACTATCGGTGCGGTATTCCATGAAGAGTACACACGAGATGGTCAATTTCAGTTATTTACAATCACGAAAGGAGGATAACCATA‑‑TGTGTTGGGCAGCCGCAATACCTATcgctatatctggcgctatatctggcgctcaggctatcagtggtcagaacgctcaggccaaaatgatcgccgcccagaccgctgctggtcgtcctcaagctctggaaatcatgaggcagacgaacatcaagaatgctgaccca                                                                                                                                                                >  1:411795‑M1/1‑159 (MQ=255)`  
                                                                                                                                                  `ggAATTACGTATGGGTAGGCAATACGTCCCACATTTGTTTCCTAAAGACTATCGGTGCGGTATTCCATGAAGAGTACACACGAGATGGTCAATTTCAGTTATTTACAATCACGAAAGGAGGATAACCATA‑‑TGTGTTGGGCAGCCGCAATACCTATcgctatatctggcgctatatctggcgctcaggctatcagtggtcagaacgctcaggccaaaatgattgccgctcagaccgctgctggtcgtcgtcaagctatggaaatcatgaggcagacgaacatccagaatgctgacctatcgt                                                                                                                                                            <  1:406002‑M1/301‑147 (MQ=255)`  
                                                                                                                                                   `gAATTACGTATGGGTAGGCAATACGTCCCACATTCGTTTCCTCAAGACTATCGGTGCGGTATTCCATGAAGAGTACACACGAGATGGTCAATTTCAGTTATTTACAATCACGAAAGGAGGATAACCATA‑‑TGTGTTGGGCAGCCGCAATACCTATcgctatatctggcgctatatctggcgctcaggctatcagtggtcagaacgctcaggccaaaatgattgccgctcagaccgctgctggtcgtcgtcaagctatggaaatcatgaggcagacgaacatccagaatgctgacctatcgtt                                                                                                                                                           >  1:93538‑M1/1‑154 (MQ=255)`  
                                                                                                                                                      `ttACGTATGGGTAGGCAATACGTCCCACATTCGTTTCCTCAAGACTATCGGTGCGGTATTCCATGAAGAGTACACACGAGATGGTCAATTTCAGTTATTTACAATCACGAAAGGAGGATAACCATA‑‑TGTGTTGGGCAGCCGCAATACCTATcgctatatctggcgctatatctggcgctcaggctatcagtggtcagaacgctcaggccaaaatgattgccgctcagaccgctgctggtcgtcgtcaagctatggaaatcatgaggcagacgaacatccagaatgctgacctatcgttgca                                                                                                                                                        <  1:243145‑M1/301‑151 (MQ=255)`  
                                                                                                                                                                `ggaggCAAAACGTCCCACATTCGTTTCCTCAAGACTATCGGTGCGGTATTCCATGAAGAGTACACACGAGATGGTCAATTTCAGTTATTTACAATCACGAAAGGAGGATAACCATA‑‑TGTGTTGGGCAGCCGCAATACCTATcgctatatctggcgctatatctggcgctcaggctatcagtggtcagaacgctcaggccaaaatgattgccgctcagaccgctgctggtcgtcgtcaagctatggaaatcatgaggcagacgaacatccagaatgctgacctatcgttgcaagctcgaagt                                                                                                                                              <  1:141590‑M1/299‑161 (MQ=255)`  
                                                                                                                                                                  `aGGCAATACGTCCCACATTCGTTTCCTCAAGACTATCGGTGCGGTATTCCATGAAGAGTACACACGAGATGGTCAATTTCAGTTATTTACAATCACGAAAGGAGGATAACCATA‑‑TGTGTTGGGCAGCCGCAATACCTATcgctatatctggcgctatatctggcgctcaggctatcagtggtcagaacgctcaggccaaaatgattgccgctcagaccgctgctggtcgtcgtcaagctatggaaatcatgaggcagacgaacatccagactgctgacctatcgtttcaagatcgaagtaa                                                                                                                                            >  1:42707‑M1/1‑139 (MQ=255)`  
                                                                                                                                                                   `ggCAATACGTCCCACATTCGTTTCCTCAAGACTATCGGTGCGGTATTCCATGAAGAGTACACACGAGATGGTCAATTTCAGTTATTTACAATCACGAAAGGAGGATAACCATA‑‑TGTGTTGGGCAGCCGCAATACCTATcgctatatctggcgctatatctggcgctcaggctatcagtggtcagaacgctcaggccaaaatgattgccgctcagaccgctgctggtcgtcgtcaagctatggaaatcatgaggcagacgaacatccagaatgctgacctatcgttgcaagctcgaagtaaa                                                                                                                                           >  1:445663‑M1/1‑138 (MQ=255)`  
                                                                                                                                                                       `aTACGTCCCACATTCGTTTCCTCAAGACTATCGGTGCCGTATTCCATGAAGAGTACACACGAGATGGCCAATTTCAGTTATTTACAATCACGAAAGCAGGTTAACCATA‑‑TGTGTTGGGCAGCCGCCATACCTATcgctatatctggcgctatatctggcgctcaggctatcagtggtcagaacactcaggccaaaatgattgcctctcagaccgctgctggtcgtcgtcaagctatggaaatcatgaggcagacgaacctccagaatgctgacctctccgtgcaaagtcgaagtaaaattt                                                                                                                                       >  1:642007‑M1/1‑134 (MQ=255)`  
                                                                                                                                                                         `aCGTCCCACATTCGTTTCCTCAAGACTATCGGTGCGGTATTCCATGAAGAGTACACACGAGATGGTCAATTTCAGTTATTTACAATCACGAAAGGAGGATAACCATA‑‑TGTGTTGGGCAGCCGCAATACCTATcgctatatctggcactatatctggcgctcaggctatcagtggtcagaacgctcaggccaaaatgattgccgctcagaccgctgctggtcgtcgtcaagctatggaaatcatgaggcagacgaacatccagaatgctgacctatcgttgcaagctcgaagtaaacttgag                                                                                                                                     <  1:506447‑M1/301‑170 (MQ=255)`  
                                                                                                                                                                             `cccACATTCGTTTCCTCAAGACGATCGGTGCGGTATTCCAGGAAGAGTACACACGAGATGGTCAATTTCAGTTATTTACAATCACGAAAGGAGGATAACCATA‑‑TGTGTTGGGCAGCAGCAATACCTATcgctatatctggcgctatatctggcgctcaggctatcagtggtcagaacgctcaggccaaaatgattgccgctcagacagctgctggtcgtcgtcaagctatggaaatcatgaggcagacgaacatccagaatgctgacctatcgttgcaagctcgaagtaaacttgaggaag                                                                                                                                 <  1:165593‑M1/301‑174 (MQ=255)`  
                                                                                                                                                                                    `tataagagacaggactatcggtgcgGTATTCCATGAAGAGTACACACGAGATGGTCAATTTCAGTTATTTACAATCACGAAAGGAGGATAACCATA‑‑TGTGTTGGGCAGCCGCAATACCTATcgctatatctggcgctatatctggcgctcaggctatcagtggtcagaacgctcaggccaaaatgattgccgctcagaccgctgctggtcgtcgtcaagctatggaaatcatgaggcagacgaacatccagaatgctgacctatcgttgcaagctcgaagtaaacttgaggaagcgtccgc                                                                                                                          <  1:153921‑M1/289‑181 (MQ=255)`  
                                                                                                                                                                                         `tCCTCAAGACTATCGGTGCGGTATTCCATGAAGAGTACACACGAGATGGTCAATTTCAGTTATTTACAATCACGAAAGGAGGATAACCATA‑‑TGTGTTGGGCAGCCGCAATACCTATcgctatatctggcgctatatctggcgctcaggctatcagtggtcagaacgctcaggccaaaatgattgccgctcagaccgctgctggtcgtcgtcaagctatggaaatcatgaggcagacgaacatccagaatgctgacctatcgttgcaagctcgaagtaaacttgaggaagcgtccgccgagt                                                                                                                     <  1:297050‑M1/301‑186 (MQ=255)`  
                                                                                                                                                                                          `ccTCAAGACTATCGGTGCGGTATTCCATGAAGAGTACACACGAGATGGTCAATTTCAGTTATTTCCAATCCCAAAAGGAGGATAACCATA‑‑TGTTTTGGGCAGCCGCAATACCTAAcgctatatctggcgctatatctggcgctcaggctatcagtgttcagaacgctcaggccaaaatgattgccgctcagaccgctgctggtcttcgtcaagctatggaaatcatgcggcagccgaacatcccaaatgctgacctatcgttgcaagctcgaagtaaacttgaggaaacgtccgccaggtt                                                                                                                    >  1:196109‑M1/1‑115 (MQ=255)`  
                                                                                                                                                                                          `ccTCAAGACTATCGGTGCGGTATTCCATGAAGAGTACACACGAGATGGTCAATTTCAGTTATTTACAATCACGAAAGGAGGATAACCATA‑‑TGTGTTGGGCAGCCGCAATACCTATcgctatatctggcgctatatctggcgctcaggctatcagtggtcagaacgctcaggccaaaatgattgccgctcagaccgctgctggtcgtcgtcaagctatggaaatcatgaggcagaagaacatccagaatgctgacctatcgttgcaagctcgacgtaaacttgaggactgtctcttaaacac                                                                                                                    >  1:592796‑M1/1‑115 (MQ=255)`  
                                                                                                                                                                                              `aaGACTATCGGTGCGGTATTCCATGAAGAGTACACACGAGATGGTCAATTTCAGTTATTTACAATCACGAAAGGAGGATAACCATA‑‑TGTGTTGGGCAGCCGCAATACCTATcgctatatctggcgctatatctggcgctcaggctatcagtggtcagaacgctcaggccaaaatgattgccgctcagaccgctgctggtcgtcgtcaagctatggaaatcatgaggcagacgaacatccagaatgctgacctatcgttgcaagctcgaagtaaacttgaggaagcgtccgccgagttgacc                                                                                                                <  1:572110‑M1/301‑191 (MQ=255)`  
                                                                                                                                                                                               `aGACTATCGGTGCGGTATTCCATGAAGAGTACACACGAGATGGTCAATTTCAGTTATTTACAATCACGAAAGGAGGATAACCATA‑‑TGTGTTGGGCAGCCGCAATACCTATcgctatatctggcgctatatctggcgctcaggctatcagtggtcagaacgctcaggccaaaatgcttgccgctcagaccgctgctggtcgtcgtcaagctatggaaatcatgaggcagacgaacatccagaatgctgacctatcgttgcaagctcgaagtaaactcgaggaagcgtccgccgagttgcccc                                                                                                               >  1:74704‑M1/1‑110 (MQ=255)`  
                                                                                                                                                                                                     `tCGGTGCGGTATTCCATGAAGAGTACACACGAGATGGTCAATTTCAGTTATTTACAATCACGAAAGGAGGATAACCATA‑‑TGTGTTGGGCAGCCGCAATACCTATcgctatatctggcgctatatctggcgctcaggctatcagtggtcagaacgctcaggccaaaatgattgccgctcagaccgctgctggtcgtcgtcaagctatggaaatcatgaggcagacgaacatccagaatgctgacctatcgttgcaagctcgaagtaaacttgaggaagcgtccgccgagttgaccccccaga                                                                                                         >  1:578397‑M1/1‑104 (MQ=255)`  
                                                                                                                                                                                                        `gTGCGGTATTCCATGAAGAGTACACACGAGATGGTCAATTTCAGTTATTTACAATCACGAAAGGAGGATAACCATA‑‑TGTGTTGGGCAGCCGCAATACCTATcgctatatctggcgctatatctggcgctcaggctatcagtggtcagaacgctcaggccaaaatgattgccgctcagaccgctgctggtcgtcgtcaagctatggaaatcatgaggcagacgaacatccagaatgctgacctatcgttgcaagctcgaagtaaacttgcggaagcgtccgccgagttgcacccacagaaca                                                                                                      >  1:370325‑M1/1‑101 (MQ=255)`  
                                                                                                                                                                                                        `gTGCGGTATTCCATGAAGAGTACACACGAGATGGTCAATTTCAGTTATTTACAATCACGAAAGGAGGATAACCATA‑‑TGTGTTGGGCAGCCGCAATACCTATcgctatatctggcgctatatctggcgctcaggctatcagtggtcagaacgctcaggccaaaatgattgccgctcagaccgctgctggtcgtcgtcaagctatggaaatcatgaggcagacgaacatccagaatgctgacctatcgttgcaagctcgaagtaaacttgaggaagcgtccgccgagttgaccccacagaaca                                                                                                      >  1:191330‑M1/1‑101 (MQ=255)`  
                                                                                                                                                                                                             `tgtataagagacaggtacacacgagatggTCAATTTCAGTTATTTACAATCACGAAAGGAGGATAACCATA‑‑TGTGTTGGGCAGCCGCAATACCTATcgctatatctggcgctatatctggcgctcaggctatcagtggtcagaacgctcaggccaaaatgattgccgctcagaccgctgctggtcgtcgtcaagctatggaaatcatgaggcagacgaacatccagaatgctgacctatcgttgcaagctcgaagtaaacttgaggaagcgtccgccgagttgaccccacagaacatgcag                                                                                                 <  1:432178‑M1/287‑206 (MQ=255)`  
                                                                                                                                                                                                             `gTATTCCATGAAGAGTACACACGAGATGGTCAATTTCAGTTATTTACAATCACGAAAGGAGGATAACCATA‑‑TGTGTTGGGCAGCCGCAATACCTATcgctatatctggcgctatatctggcgctcaggctatcagtggtcagaacgctcaggccaaaatgattgccgctcagaccgctgctggtcgtcgtcaagctatggaaatcatgaggcagacgaacatccagaatgctgacctatcgttgcaagctcgaagtaaacttgaggaagcgtccgccgagttgaccccacagaacatgcag                                                                                                 >  1:566401‑M1/1‑96 (MQ=255)`  
                                                                                                                                                                                                               `aTTCCATGAAGAGTACACACGAGATGGTCAATTTCAGTTATTTACAATCACGAAAGGAGGATAACCATA‑‑TGTGTTGGGCAGCCGCAATACCTATcgctatatctggcgctatatctggcgctcaggctatcagtggtcagaacgctcaggccaaaatgattgccgctcagaccgctgctggtcgtcgtcaagctatggaaatcatgaggcagacgaacatccagaatgctgacctatcgttgcaagctcgaagtaaacttgaggaagcgtccgccgagttgaccccacagaacatgcagaa                                                                                               >  1:225961‑M1/1‑94 (MQ=255)`  
                                                                                                                                                                                                                `agccatGAAGAGTACACACGAGATGGTCAATTTCAGTTATTTACAATCACGAAAGGAGTATAACCATA‑‑TGTGTTGGGCAGCCGCAATACCTATcgctatatctggcgctatatctggcgctcaggctatcagtggtcagaacgctcaggccaacatgattgccgctcagaccgctgctggtcgtcgtcaagctatggaaatcatgaggcagacgaacatccagaatgctgacctatcgttgcaagctcgaagtaaacttgaggaagcgtccgccgagttgaccccacagaacatgcagaag                                                                                              <  1:219335‑M1/299‑209 (MQ=255)`  
                                                                                                                                                                                                                 `tCCATGAAGAGTACACACGAGATGGTCAATTTCAGTTATTTACAATCACGAAAGGAGGATAACCATA‑‑TGTGTTGGTCAGCCGCAATACCTATcgctatatctggcgctatatctggcgctcaggctatcagtggtcagaacgctcaggccaaaatgattgccgctccgaccgctgctggtcgtcgtcaagctatggaaatcatgaggcagccaaacatcccgaatgctgacctatcgttgcaagctcgaagtaacctttaggcagcaccccccgagtttaccccacagaacatgcagaagg                                                                                             >  1:663181‑M1/1‑92 (MQ=255)`  
                                                                                                                                                                                                                 `tCCATGAAGAGTACACACGAGATGGTCAATTTCAGTTATTTACAATCACGAAAGGAGGATAACCATA‑‑TGTGTTGGGCAGCCGCAATACCTATcgctatatctggcgctatatctggcgctcaggctatcagtggtcagaacgctcaggccaaaatgattgccgctcagaccgctgctggtcgtcgtcaagctatggaaatcattaggcagacgaacatccagaatgctgacctatcgttgcaagctcgcagtaaacttgaggaagcgtccgccgagttgaccccacagaacatgcagcatg                                                                                             >  1:561155‑M1/1‑92 (MQ=255)`  
                                                                                                                                                                                                                 `tCCATGAAGAGTACACACGAGATGGTCAATTTCAGTTATTTACAATCACGAAAGGAGGATAACCATA‑‑TGTGTTGGGCAGCCGCAATACCTATcgctatatctggcgctatatctggcgctcaggctatcagtggtcagaacgctcaggccaaaatgattgccgctcagaccgctgctggtcgtcgtcaagctatggaaatcatgaggcagacgaacatccagaatgctgacctatcgttgcaagctcgaagtaaacttgaggaagcgtccgccgagttgaccccacagaacatgcagaagg                                                                                             >  1:636598‑M1/1‑92 (MQ=255)`  
                                                                                                                                                                                                                 `tCCATGAAGAGTACACACGAGATGGTCAATTTCAGTTATTTACAATCACGAAAGGAGGATAACCATA‑‑TGTGTTGGGCAGCCGCAATACCTATcgctatatctggcgctatatctggcgctcaggctatcagtggtcagaacgctcaggccaaaatgattgccgctcagaccgatgctggtcgtcgtcaagctatggaaatcatgaggcagacgaacatccagaatgctgacctatcgttgcaagctcgaagtaaacttgaggaagcgtccgccgagttgaccccacagaacatgcagaagg                                                                                             >  1:179448‑M1/1‑92 (MQ=255)`  
                                                                                                                                                                                                                 `tCCATGAAGAGTACACACGAGATGGTCAATTTCAGTTATTTACAATCACGAAAGGAGGATAACCATA‑‑TGTGTTGGGCAGCCGCAATACCTATcgctatatctggcgctatatctggcgctcaggctatcagtggtcagaacgctaaggccaaaatgattgccgctcagaccgctgctggtcgtcgtcaagctatggaaatcatgaggcagacgaacatccagaatgctgacctatcgttgcaagctcgaagtaaacttgaggaagcgtccgccgagttgaccccacagaacatgcagaagg                                                                                             >  1:494678‑M1/1‑92 (MQ=255)`  
                                                                                                                                                                                                                      `gAAGAGTACACACGAGATGGTCAATTTCAGTTATTTACAATCACGAAAGGAGGATAACCATA‑‑TGTGTTGGGCAGCCGCAATACCTATcgctatatctggcgctatatctggcgctcaggctatcagtggtcagaacgctcaggccaaaatgattgccgctcagaccgctgctggtcgtcgtcaagctatggaaatcatgaggcagacgaacatccagaatgctgacctatcgttgcaagctcgaagtcaacttgaggaagcgtccgccgagttgaccccacagaacatgcagaagctccaa                                                                                        >  1:438596‑M1/1‑87 (MQ=255)`  
                                                                                                                                                                                                                       `aaGAGTACACACGAGATGGTCAATTTCAGTTATTTACAATCACGAAAGGAGGATAACCATA‑‑TGTGTTGGGCAGCCGCAATACCTATcgctatatctggcgctatatctggcgctcaggctatcagtggtcagaacgctcaggccaaaatgattgccgctcagaccgctgctggtcgtcgtcaagctatggaaatcatgaggcagacgaacatccagaatgctgacctatcgttgcaagctcgaagtaaacttgaggaagcgtccgccgagttgaccccacagaacatgcagaaggtccaag                                                                                       <  1:399295‑M1/301‑216 (MQ=255)`  
                                                                                                                                                                                                                             `acacacGAGATGGTCAATTTCAGTTATTTACAATCACGAAAGGAGGATAACCATA‑‑TGTGTTGGGCAGCCGCAATACCTATcgctatatctggcgctatatctggcgctcaggctatcagtggtcagaacgctcaggccaaaatgattgccgctcagaccgctgctggtcgtcgtcaagctatggaaatcatgaggcagacgaacatccagaatgctgacctatcgttgcaagctcgaagtaaacttgaggaagcgtccgccgagttgacctcacagaacatgcagaaggtccaagctattg                                                                                 <  1:500879‑M1/301‑222 (MQ=255)`  
                                                                                                                                                                                                                              `cacacGAGCTGGTCAATTTCAGTTATTTACAATCACGAAAGGAGTATAACCATA‑‑TGTGTTGGGCAGCCGCAATACCTATcgctatatctggcgctatatctggcgctcaggctatcagtggtcagaacgctcaggccaaaatgattgccgctcagaccgctgctggtcgtcgtcaagctatggaaatcatgaggcagacgaacatccagaatgctgacctatcgttgcaagctcgaagtaaacttgaggaagcgtccgccgagttgaccccacagaacatgcagaaggtccaagctattgg                                                                                >  1:340578‑M1/1‑79 (MQ=255)`  
                                                                                                                                                                                                                              `cacacGAGATGGTCAATTTCAGTTATTTACAATCACGAAAGGAGGATAACCATA‑‑TGTGTTGGGCAGCCGCAATACCTATcgctatatctggcgctatatctggcgctcaggctatcagtggtcagaacgctcaggccaaaatgattgccgctcagaccgctgctggtcgtcgtcaagctatggaaatcatgaggcagacgaacatccagaatgctgacctatcgttgcaagctcgaagtaaacttgaggaagcgtccgccgagttgaccccacagaacatgcagaaggtccaagctattgg                                                                                >  1:269060‑M1/1‑79 (MQ=255)`  
                                                                                                                                                                                                                                `cacGAGATGGTCAAGTTCAGTTATTTACAATCACGAAAGGAGGATAACCATA‑‑TGTGTTGGGCAGCCGCAATACCTATcgctatatctggcgctatatctggcgctcaggctatcagtggtcagaacgctcaggccaaaatgattgccgctcagaccgctgctggtcgtcgtcaagctatggaaatcatgaggcagacgaacatccagaatgctgacctatcgttgcaagctcgaagtaaacttgaggaagcgtccgccgagttgacctcacagaacatgcagaaggtccaagctattgggt                                                                              <  1:451502‑M1/301‑225 (MQ=255)`  
                                                                                                                                                                                                                                `aacgATATGGTCAATTTCAGTTATTTACAATCACGAAAGGAGGATAACCATA‑‑TGGGTTGGGCAGCCGCAATACCTATcgctatatctggcgctatatctggcgctcaggctagcagtggtcagaacgctcaggccaaaatgattgccgctcagaccgctgctggtcgtcgtcaagctatggaaatcatgaggcagacgaacatccagaatgctgacctatcgttgcaagctcgaagtaaactggaggaagcgtccgccgagttgaccccacagaacatgcagaaggtccaagctattgggt                                                                              <  1:629834‑M1/300‑225 (MQ=255)`  
                                                                                                                                                                                                                                `aacgAGATGGTCAATTTCAGTTATTTACAATCACGAAAGGAGGATAACCATA‑‑TGTGTTGGGCAGCCGCAATACCTATcgctatatctggcgctatatctggcgctcaggctatcagtggtcagtacgctcaggccaaaatgattgccgctcagaccgctgctggtcgtcgtcaagctatggaaatcatgaggcagacgaacatccagaatgctgacctatcgttgcaagctcgaagtaaacttgaggaagcgtccgccgagttgaccccacagaacatgcagaaggtccaagctattgggt                                                                              <  1:232002‑M1/300‑225 (MQ=255)`  
                                                                                                                                                                                                                                 `acGAGATGGTCAATTTCAGTTATTTACAATCACGAAAGGAGGATAACCATA‑‑TGTGTTGGGCAGCCGCAATACCTATcgctatatctggcgctatatctggcgctcaggctatcagtggtcagaacgctcaggccaaaatgattgccgctcagaccgctgctggtcgtcgtcaagctatggaaatcatgaggcagacgaacatccagaatgctgacctatcgttgcaagctcgaagtaaacttgaggaagcgtccgccgagttgaccccacagaacatgcagaaggtccaagctattgggtc                                                                             <  1:555501‑M1/301‑226 (MQ=255)`  
                                                                                                                                                                                                                                 `acGAGATGGTCAATTGCAGTTATTTACAATCACGAAAGGAGGATAACCATA‑‑TGTGTTGGGCAGCCGCAATACCTATcgctatatctggcgctatatctggcgctcaggctatcagtggtcagaacgctcaggccaaaatgattgccgctcagaccgctgctggtcgtcgtcaagctatggaaataatgaggcagacgaacaaccagaatgctgacctatcgttgcaagctcgaagtaaacttgaggaagcgtccgccgagttgacctcacagaacatgcagaaggtccaagctattgggtc                                                                             <  1:653620‑M1/301‑226 (MQ=255)`  
                                                                                                                                                                                                                                     `gaTTGTCAATTGCAGTGATTTACAATCACGAAAGGAGGATAACCATA‑‑TGTGTGGGGCAGCCGCAATACCTATcgctatatctggcgctatatctggcgctcaggctatcagtggtcagaacgctcaggccaaaatgagtgccgctcagaccgcttctggtcgtcgtcaagctatggaaatcatgatgcagacgaacatccagaatgctgacctatcgttgcaagctcgaagtaaacgtgaggaagcgtccgccgagttgaccccacagaacatgcagaaggtccaagctattgggtctatc                                                                         <  1:237290‑M1/301‑230 (MQ=255)`  
                                                                                                                                                                                                                                             `aTTTCAGTTATTTACAATCACGAAAGGAGGATAACCATA‑‑TGTGTTGGGCAGCCGCAATACCTATcgctatatctggcgctatatctggcgctcaggctatcagtggtcagaacgctcaggccaaaatgattgccgctcagaccgctgctggtcgtcgtcaagctatggaaatcatgaggcagacgaacatccagaatgctgacctatcgttgcaagctcgaagtaaacttgaggaagcgtccgccgagtttaccccaccgaacctgcagaaggtccaagctattgggtctatccgcacggc                                                                 >  1:313994‑M1/1‑64 (MQ=255)`  
                                                                                                                                                                                                                                                           `cAATCACGAAAGGAGGATAACCATA‑‑TGTGTTGGGCAGCCGCCATACCTATcgctatatctggcgctatatctggcgctcaggctatcagtggtcagaacgctcaggccaaaatgattgccgctcagaccgctgctggtcgtcgtcaagctatggaaatcatgaggcagacgaacatccagaatgctgacctatcgttgcaagctcgaagtaaacttgaggaagcgtccgccgagttgaccccacagaacatgcagaaggtccaagctattgggtctatccgagcggctatcggagagagta                                                   <  1:536708‑M1/301‑252 (MQ=255)`  
                                                                                                                                                                                                                                                              `aagagacaggaggataaccaTA‑‑TGTGTTGGGCAGCCGCAATACCTATcgctatatctggcgctatatctggcgctcaggctatcagtggtcagaacgctcaggccaaaatgattgccgctcagaccgctgctggtcgtcgtcaagctatggaaatcatgaggcagacgaacatccagaatgctgacctatcgttgcaagctcgaagtaaacttgaggaagcgtccgccgagttgaccccacagaacatgcagaaggtccaagctattgggtctatccgagcggctatcggagagagtatgc                                                <  1:159406‑M1/294‑255 (MQ=255)`  
                                                                                                                                                                                                                                                               `cACGAAAGGAGGATAACCATA‑‑TGTGTTGGGCAGCCGCAATACCTATcgctatatctggcgctatatctggcgctcaggctatcagtggtcagaacgctcaggccaaaatgattgtcgctcagaccgctgctggtcgtcgtcaagctatggaaatcatgaggcagacgaacatctagaatgctgacctatcgttgcaagctcgaagtaaacttgaggaagcgtccgccgagttgaccccacagaacatgcagaaggtccaagctattgggtctatccgagcggctatcggagagagtatgct                                               <  1:586770‑M1/301‑256 (MQ=255)`  
                                                                                                                                                                                                                                                               `cACGAAAGGAGGATAACCATA‑‑TGTGTTGGGCAGCCGCAATACCTATcgctatatctggcgctatatctggcgctcaggctatcagtggtcagaacgcgcaggccaaaaggattgccgctcagaccgctgctggtcgtcgtcaagctatggaaatcatgaggcagacgaacatccagaatgctgacctatcgttgcaagcgcgaagtaaacttgaggaagcgtccgccgagttgaccacacagaacatgcagaaggtccaagctattgggtctatccgagcggctatcggagagagtatgct                                               <  1:120225‑M1/301‑256 (MQ=255)`  
                                                                                                                                                                                                                                                                 `cGAAAGGAGGATAACCATA‑‑TGTGTTGGGCAGCCGCAATACCTATcgctatatctggcgctatatctggcgctcaggctatcagtggtcagaacgctcaggccaaaatgattgccgctcagaccgctgctggtcgtcgtcaagctaaggaaatcatgcggcagacgaacatccagaatgctgacctatcgttgcaagctcgaagtaaacttgcggatgcgtccgccgagttgaccccacagcacatgcacacggtccaatcccttggttctatcctaccgcctatcatagagatgctgctcg                                             >  1:375862‑M1/1‑44 (MQ=255)`  
                                                                                                                                                                                                                                                                    `aaGGAGGATAACCATA‑‑TGTGTTGGGCAGCCGCAATACCTATcgctatatctggcgctatatctggcgctcaggctatcagtggtcagaacgctcaggccaaaatgattgccgctcagaccgctgctggtcgtcgtcaagctatggaaatcatgaggcagacgaacatccagaatgctgacctatcgttgcaagctcgaagtaaacttgaggaagcgtccgccgagttgaccccacagaacatgcagaaggtccaagctattgggtctatccgagcggctatcggagagagtatgcttgaag                                          <  1:345331‑M1/301‑261 (MQ=255)`  
                                                                                                                                                                                                                                                                     `aggaggATAACCATA‑‑TGTGTTGGGCAGCCGCAATACCTATcgctatatctggcgctatatctggcgctcaggctatcagtggtcagaacgctcaggccaaaatgattgccgctcagaccgctgctggtcgtcgtcaaactatggaaatcatgaggcagacgaacatccagaatgctgacctatcgttgcaagctcgaagtaaacttgaggaagcgtccgccgagttgaccccacagaacatgcagaaggtccaagctattgggtctatccgagcggctatcggagagagtatgcttgaagg                                         >  1:291089‑M1/1‑40 (MQ=255)`  
                                                                                                                                                                                                                                                                     `aggaggATAACCATA‑‑TGTGTTGGGCAGCCGCAATACCTATcgctatatctggcgctatatctggcgctcaggctatcagtggtcagaacgctcaggccaaaatgattgccgctaagaccgctgctggtcgtcgtcaagctatggaaatcatgaggcagacgaacatccagaatgctgacctatcgttgcaagctcgaagtaaacttgaggaagcgtccgccgagttgaccccacagaacatgcagaaggtccaagctattgggtctatccgagcggctatcggagaaagtatgcttgaagg                                         <  1:604456‑M1/301‑262 (MQ=255)`  
                                                                                                                                                                                                                                                                     `aggaggATAACCATA‑‑TGTGTTGGGCAGCCGCAATACCTATcgctatatctggcgctacatctggcgctcaggctatcagtggtcaaaacgctcaggccaaaatgattgccgctcagaccgctgctggtcgtcgtcaagctatggaaatcatgaggcagacgaacatccagaatgctgacctatcgttgcaagctcgaagtaaacttgatgaagcgtccgccgagttgaccacacagaacatgcagaaggtccaagctattgggtctatccgagcggctatcggagagagtatgcttgaagg                                         <  1:255028‑M1/301‑262 (MQ=255)`  
                                                                                                                                                                                                                                                                       `gaggaTAACCATA‑‑TGTGTTGGGCAGCCGCAATACCTATcgctatatctggcgctatatctggcgctcaggctatcagtggtcagaacgctcaggccaaaatgattgccgctcagaccgctgctggtcgtcgtcaagctatggaaatcatgaggcagacgaacatccagaatgctgacctatcgttgcaagctcgaagtaaacttgaggaagcgtccgccgagttgaccccacagaacatgcagaaggtccaagctattgggtctatccgagcggctatcggagagagtatgcttgaaggtt                                       >  1:524072‑M1/1‑38 (MQ=255)`  
                                                                                                                                                                                                                                                                         `ggaTAACCATA‑‑TGTGTTGGGCAGCCGCAATACCTATcgctatatctggcgctatatctggcgctcaggctatcagtggtcagaacgctcaggccaaaatgattgccgctcagaccgctgctggtcgtcgtcaagctatggaaatcatgaggcagacgaacatccagaatgctgacctatcgttgcaagctcgaagtaaacttgaggaagcgtccgccgagttgaccccacagaacatgcagaaggtccaagctattgggtctatccgagcggctatcggagagagtatgcttgaaggttcc                                     >  1:213321‑M1/1‑36 (MQ=255)`  
                                                                                                                                                                                                                                                                             `aaCCATA‑‑TGTGTTGGGCAGCCGCAATACCTATcgctatatctggcgctatatctggcgctcaggctatcagtggtcagaacgctcaggccaaaatgattgccgctcagaccgctgctggtcgtcgtcaagctatggaaatcatgaggcagacgaacatccagaatgctgacctatcgttgcaagctcgaagtaaacttgaggaagcgtccgccgagttgaccccacagaacatgcagaaggtccaagctattgggtctatccgagcggctatcggagagagtactgtctcttatacacatc                                 >  1:112934‑M1/1‑32 (MQ=255)`  
                                                                                                                                                                                                                                                                               `tcaTA‑‑TGTGTTGGGCAGCCGCAATACCTATcgctatatctggcgctatatctggcgctcaggctatcagtggtcagaacgctcaggccaaaatgattgccgctcagaccgctgctggacgtcgtcaagctatggaaatcatgaggcagacgaacatccagaatgctgacctatcgttgcaagctcgaagtaaacttgaggaagcgtccgccgagttgaccccacagaacatgcagaaggtccaagctattgggtctatccgagcggctatcggagagagtatgcttgaaggttcctcaatg                               >  1:623055‑M1/2‑30 (MQ=255)`  
                                                                                                                                                                                                                                                                               `ggctcggagatgtgtataagatacagacctatcgctatatctggcgctatatctggcgctcaggcgagaagtggtcagaacgctcaggacaaaatgattgacgctcagaccgctgctgggcgtcgtcaagctatggaaatcatgaggcagacgaacatccagaatgctgacctatcggtgcaagcgcgaaggaaacttgaggaagcgtccgccgagttgaccccacagaacatgcagaaggtacaagctattgggtctatccgagcggctatcggagagagtatgcttgaaggttcctcaa                                 <  1:363042‑M1/275‑270 (MQ=255)`  
                                                                                                                                                                                                                                                                                `cATA‑‑TGTGTTGGGCAGCCGCAATACCTATcgctatatctggcgctatatctggcgctcaggctatcagtggtcagaacgctcaggccaaaatgattgccgctcagaccgctgctggtcgtcgtcaagctatggaaatcatgaggcagacgaacatccagaatgctgacctatcgttgcaagctcgaagtaaacttgaggaagcgtccgccgagttgaccccacagaacatgcagaaggtccaagctattgggtctatccgagcggctatcggagagagtatgcttgaaggttcctcaatgg                              <  1:135182‑M1/301‑273 (MQ=255)`  
                                                                                                                                                                                                                                                                                       `gtgtTGGGCAGCCGCAATACCTATcgctatatctggcgctatatctggcgctcaggctatcagtggtcagaacgctcaggccaaaatgattgccgctcagaccgctgctggtcgtcgtcaagctatggaaatcatgaggcagacgaacatccagaatgctgacctatcgttgcaagctcgaaggaaacttgaggaagcgtccgccgagttgaccccacagaacatgcagaaggtccaagctagtgggtcgatcagagcggctatcggagagaggatgcttgaaggttcctcaatgacccgc                         >  1:207285‑M1/1‑24 (MQ=255)`  
                                                                                                                                                                                                                                                                                       `gtgtTGGGCAGCCGCAATACCTATcgctatatctggcgctatatctggcgctcaggctatcagtggtcagaacgctcaggccaaaatgattgccgctcagaccgctgctggtcgtcgtcaagctatggaaatcatgaagcagacgaacatccagaatgctgacctatcgttgcaagctcgaagtaaacttgaggaagcgtccgccgagttgaccccacagaacatgcagaaggtccaagctattgggtctatccgagcggctatcggagcgagtatgcttgaaggttcctcaatggctgtt                         >  1:285243‑M1/1‑24 (MQ=255)`  
                                                                                                                                                                                                                                                                                       `gtgtTCGGCAGCCGCAATACCTATcgctatatctggcgctatatctggccctcaggctatcagtggtcagaacgcttaggccaaaatgattgccgctcagaccgctgctggtcgtcgccaagctatggaaatcatgaggcagacgaacatccaacatgctgacctatcgttgcaagctcgaagtaaacttgaggaagcgtccgccgcgttgaccccacagaacatgaagaaggtccaagcgattgtgtctacacgagagactatccgagcgactatgcttgaagggtcctccattgacagc                         >  1:202076‑M1/1‑24 (MQ=255)`  
                                                                                                                                                                                                                                                                                              `gCAGCCGCAATACCTATcgctatatctggcgctatatctggcgctcaggctatcagtggtcagaacgctcaggccaaaatgattgccgctcagaccgctgctggtcgtcgtcaagctatggaaatcatgaggcagacgaacatccagaatgctgacctatcgttgcaagctcgaagtaaacttgaggaagcgtccgccgagttgaccccacagaacatgcagaaggtccaagctattgggtctatccgagcggctatcggagagagtatgcttgaaggttcctcaatggaccgcattaagc                  <  1:513188‑M1/301‑285 (MQ=255)`  
                                                                                                                                                                                                                                                                                              `gCAGCCACAATACCTATcgctatatctggcgctatatctggcgctcaggctatcagtggtcagaacgctcaggccaaaatgattgccgctcagaccgctgctggtcgtcgtcaagctatggaaatcatgaggcagacgaacatccagaatgctgacctatcgttacaagctcgaagtaaacttgaggaagagtccgccgagttgaccccacagaacatgcagaaggtccaagctattgggtctatccgagcggctatcggagagagtatgcttgaaggttcctcaatggaccgcattaagc                  <  1:32312‑M1/301‑285 (MQ=255)`  
                                                                                                                                                                                                                                                                                               `cAGCCGCAATACCTATcgctatagctggcgctatatctggcgctcaggctatcagtggtcagaacgctcaggccaaaatgattgccgctcagaccgctgctggtcgtcgtcaagctatggaaatcatgaggcagacgaacatccagaatgctgacctatcgttgcaagctcgaagtaaacttgaggaagcgtccgccgagttgaccccacagaacatgcagaaggtccaagctattgggtctatccgagcggctatcggagagagtatgcttgaaggttcctcaatggaccgcattaagcg                 <  1:27976‑M1/301‑286 (MQ=255)`  
                                                                                                                                                                                                                                                                                                 `gCCGCAATACCTATcgctatatctggcgctatatctggcgctcaggctatcagtggtcagaacgctcaggccaaaatgattgccgctcagaccgctgctggtcgtcgtcaagctatggaaatcatgaggcagacgaacatccagaatgctgacctatcgttgcaagctcgaagtcaacttgaggaagcgtccgccgagttgaccccacagaacatgcagaagctccacgctattgggtctatccgagcggctatcggagagagtatgcttgaaggttcctcaatggaacgcattaagcgag               >  1:350705‑M1/1‑14 (MQ=255)`  
                                                                                                                                                                                                                                                                                                 `gCCGCAATACCTATcgctatatctggcgctatatctggcgctcaggctatcagtggtcagaacgctcaggccaaaatgattgccgctcagaccgctgctggtcgtcgtcaagctatggaaatcatgaggcagacgaacatccagaatgctgacctatcgttgcaagctcgaagtaaacttgaggaagcgtccgccgagttgaccccacagaacatgcagaaggtccaagctattgggtctatccgagcggctatcggagagagtatgcttgaaggttcctcaatggaccgcattaagcgct               >  1:24906‑M1/1‑14 (MQ=255)`  
                                                                                                                                                                                                                                                                                                    `gCAATACCTATcgctatatctggcgctatatctggcgctcaggctatcagtggtcagaacgctcaggccaaaatgattgccgctcagaccgctgctggtcgtcgtcaagctatggaaatcatgaggcagacgaacatccagaatgctgacctatcgttgcaagctcgaagtaaacttgaggaagcgtccgccgagttgaccccacagaacatgcagaaggtccaagctattgggtctatccgagtggctatcggagagagtatgcttgaaggttcctcaatggaccgcattaagcgagtaa            >  1:335922‑M1/1‑11 (MQ=255)`  
                                                                                                                                                                                                                                                                                                        `aacCTATcgctatatctggcgctatatctggcgctcaggctatcagtggtcagaacgctcaggccaaaatgattgccgctcagaccgctgctggtcgtcgtcaagctatggaaatcatgaggcagacgaacatccagaatgctgacctatcgttgcaagctcgaagtaaacttgaggaagcgtccgccgagttgaccccacagaacatgcagaaggtccaagctattgggtctatccgagcggctatcggagagagtatgcttgaaggttcctcaatggaccgcattaagcgagtcacaga        <  1:35646‑M1/300‑295 (MQ=255)`  
                                                                                                                                                                                                                                                                                                        `aacCTATcgctatagctggcgctatatctggcgctcaggctatcagttgtcagaacgataaggccaaaatgattgaagctcagaccggtgctggtcgtcgtaaagctatggaaatcatgaggcagacgaacatccagaatgctgacctatcgttgcaagctcgaagtaaacttgaggaagcgtccgccgagttgaacccacagaacatgcagaaggtccaagctattgggtctatccgagcggctatcggagagagtatgcttgaaggttcctcaatggaccgcattaagcgagtcacaga        <  1:100163‑M1/300‑295 (MQ=255)`  
                                                                                                                                                                                                                                                                                                         `aCCTATcgctatatctggcgctatatctggcgctcaggctatcagtggtcagaacgctcaggccaaaatgattgccgctcagacctctgctggtcgtcgtcacgctatggaaatcattaggcagacgaacatccagaatgctgacatatcgttgcaagctcgaagtaaactttaggaagcgtccgccgagttgaccccacagaacatgcagaaggtccaagctattgggtctatccgagcggttatcggagagcttatgcttgaaggttcctcaatggaccgcattaagcgctgtctcttt       >  1:542380‑M1/1‑6 (MQ=255)`  
                                                                                                                                                                                                                                                                                                         `aCCTATcgctatatctggcgctatatctggcgctcaggctatcagtggtcagaacgctcaggccaaaatgattgccgctcagaccgctgctggtcgtcgtcaagctatggaaatcatgaggcagacgaacatccagaatgctgacctatcgttgcaagctcgaagtaaacttgaggaagcgtccgccgagttgaccccacagaacatgcagaaggtccaagctattggggctatccgagcggctatcggagagagtatgcttgaaggttcctccatggaccgcattaagcaagtcacagaa       >  1:658894‑M1/1‑6 (MQ=255)`  
                                                                                                                                                                                                                                                                                                         `aCCTATcgctatatctggcgctatatctggcgctcaggctatcagtggtcagaacgctcaggccaaaatgattgccgctcagaccgctgctggtcgtcgtcaagctatggaaatcatgaggcagacgaacatccagaatgctgacctatcgttgcaagctcgaagtaaacttgaagaagcgtccgccgagttgacctcacagaacatgcagaaggtccaagctattgggtctatccgagcggctatcggagagagtatgcttgaaggttcctcaatggcccgcattaagcgagtccaagac       >  1:330668‑M1/1‑6 (MQ=255)`  
                                                                                                                                                                                                                                                                                                         `aCCTATcgctatatctggcgctatatctggcgctcaggctatcagtggtcagaacgctcaggccaaaatgattgcagctcagaccgctgctggtcgtcgtcaagctatggaaatcatgaggcagacgaacatccagaatgctgacctatcgttgcaagctcgaagtaaacttgaggaagcgtccgccgagttgaccccacagaacatgcagaaggtccaagctattgggtctatccgagcggctatcggagagagtatgcttgaaggttcctcaatggaccgcattaagcgctgtctctta       >  1:390001‑M1/1‑6 (MQ=255)`  
                                                                                                                                                                                                                                                                                                          `ccTAGcgctatatctggcgctatagctggcgctcaggctatcagtggtcagaacgctcaggcaaaaatgatggccgctcagaccgctgctggtcgtcgtcaagctatggaaataatgaggcagacgaacatccagaatgctgacctaacgttgcaagctagaagtaaacttgaggaagcgtccgccgagttgacccaacagaacatgcagaagggccaagctattgggtctatccgagcggctatcggagagagtatgcttgaaggttcctcaatggaccgcattaagcgagtcacagaag      <  1:395287‑M1/301‑297 (MQ=255)`  
                                                                                                                                                                                                                                                                                                             `aTcgctatatctggcgctatatctggcgctcaggctatcagtggtcagaacgctcaggccaaaatgattgccgctcagaccgctgctggtcgtcgtcaagctatggaaatcatgaggcagacgaacatccagaatgctgacctatcgttgcaagctcgaagtaaacttgaggaagcgtccgccgagttgaccccacagaacatgcagaaggtccaagctattgggtctatccgagcggctatcggagagagtatgcttgaaggttcctcaatggaccgcattaagcgagtcacagaaggac   <  1:307891‑M1/301‑300 (MQ=255)`  
                                                                                                                                                                                                                                                                                                              `tcgctatatctggcgctatatctggcgctcaggctatcagtggtcagaacgctcaggccaaaatgattgccgctcagaccgctgctggtcgtcgtcaagctatggaaatcatgaggcagacgaacatccagaatgctgacctatcgttgcaagctcgaagtaaacttgaggaagcgtccgccgagttgaccccacagaacatgcagaaggtccaagctattgggtctatccgagcggctatcggagagagtatgcttgaaggttcctccatggaccgcattaagcgagtcacagaaggcca  >  1:86592‑M1/1‑1 (MQ=255)`  

                                                                                                                                                                                                                                                                                                                                                                                                                                                                                                                                                                                                                             
`TCTAGCTATCGGTGGTAACTGCGGGGACCAGTGCTGGTTCGTTACGAGCGACCAAGTGTGGCGACTTAGTGGAAAGGCTAAGCGAAAGTTCCGTAAGTTAATCATGGAGTATCGCGATAAGATGCTTGAGAAGTATGATACTCTTTGGAATTACGTATGGGTAGGCAATACGTCCCACATTCGTTTCCTCAAGACTATCGGTGCGGTATTCCATGAAGAGTACACACGAGATGGTCAATTTCAGTTATTTACAATCACGAAAGGAGGATAACCATA‑‑TGTGTTGGGCAGCCGCAATACCTATCGCTATATCTGGCGCTCAGGCTATCAGTGGTCAGAACGCTCAGGCCAAAATGATTGCCGCTCAGACCGCTGCTGGTCGTCGTCAAGCTATGGAAATCATGAGGCAGACGAACATCCAGAATGCTGACCTATCGTTGCAAGCTCGAAGTAAACTTGAGGAAGCGTCCGCCGAGTTGACCTCACAGAACATGCAGAAGGTCCAAGCTATTGGGTCTATCCGAGCGGCTATCGGAGAGAGTATGCTTGAAGGTTCCTCAATGGACCGCATTAAGCGAGTCACAGAAGGACAGTTCATTCGGGA  >  NC_001604.1/27453‑28053`

  

Alignment Legend

Aligned base mismatch/match (shaded by quality score): `ATCG/ATCG` `< 3 ≤` `ATCG/ATCG` `< 10 ≤` `ATCG/ATCG` `< 22 ≤` `ATCG/ATCG` `< 34 ≤` `ATCG/ATCG` `< 38 ≤` `ATCG/ATCG`

Unaligned base: `atcg`    Masked matching base: `atcg`    Alignment gap: `‑`    Deleted base: `‑`
